# Experimental study of isolas in nonlinear systems featuring modal interactions

**DOI:** 10.1371/journal.pone.0194452

**Published:** 2018-03-27

**Authors:** Thibaut Detroux, Jean-Philippe Noël, Lawrence N. Virgin, Gaëtan Kerschen

**Affiliations:** 1 Space Structures and Systems Laboratory, Aerospace and Mechanical Engineering Department, University of Liège, Liège, Belgium; 2 Nonlinear Dynamics Group, Pratt School of Engineering, Duke University, Durham, North Carolina, United States of America; University of Michigan, UNITED STATES

## Abstract

The objective of the present paper is to provide experimental evidence of isolated resonances in the frequency response of nonlinear mechanical systems. More specifically, this work explores the presence of isolas, which are periodic solutions detached from the main frequency response, in the case of a nonlinear set-up consisting of two masses sliding on a horizontal guide. A careful experimental investigation of isolas is carried out using responses to swept-sine and stepped-sine excitations. The experimental findings are validated with advanced numerical simulations combining nonlinear modal analysis and bifurcation monitoring. In particular, the interactions between two nonlinear normal modes are shown to be responsible for the creation of the isolas.

## Introduction

In many fields including engineering, physics or chemistry, the concept of resonance is fundamental. A resonance, whichever the physical system it affects, results from the frequency-dependent nature of the system response. When fulfilled, resonance conditions commonly translate into dynamic oscillations with increasing amplitude as, *e.g.*, in stringed instruments, ships shaken by the ocean waves or human organs during magnetic resonance imaging. For mechanical applications in particular, special attention should be paid to the treatment of structural resonances, as they may cause excessive deformations leading to fatigue and potentially to complete collapse. Structural resonances are thus generally avoided or, if tolerated, mitigated using vibration absorbers [1, 2].

Under the assumption of linearity, the response of a system at resonance is unique, and a doubling of the input results in a doubling of the output. A structure operating in nonlinear regime of motion, on the other hand, may exhibit rich dynamical features in the vicinity of its resonances [3]. A common symptom of nonlinearity is the dependency of the resonance frequency on the energy introduced in the system, leading to hardening or softening effects and to the well-known jump phenomenon. The present paper is dedicated to the study of a much less documented nonlinear phenomenon, called isolated resonance. Isolated resonances are located at the extremities of so-called isolas, also termed islands or isolated/detached response curves, which correspond to closed loops of periodic solutions detached from the main frequency response of the nonlinear system [4].

Isolas can lie inside the main resonance peaks [5], or outside [6, 7]. Isolas and isolated resonances may thus go easily undetected, whether it be numerically employing classical continuation techniques, or experimentally. However, an increase in forcing amplitude, or the variation of a system parameter, may cause the merging of the isola with the main frequency response. The isola merging may lead to dramatic shifts of the resonance frequency and amplitude, as exemplified in [8, 9]. This renders isolas and isolated resonances potentially dangerous for nonlinear systems. They were found, for instance, to limit the practical applicability of nonlinear absorbers [10, 11]. They were also revealed in other numerical applications, such as shimmying wheels [12] and structures with cyclic symmetry [13], demonstrating their generic character.

The unicity and fairly easy predictability of linear resonances is a direct consequence of the linear normal mode (LNM) theory, which allows a decomposition of linear response into a superposition of the responses of independent and simpler oscillators [14]. The situation is contrasted in the study of nonlinear resonances, including both fundamental and isolated resonances, which require more advanced theoretical tools. The concept of nonlinear normal modes (NNMs) was proposed by Rosenberg as a straightforward nonlinear extension of the LNMs. He defined NNMs as the vibrations in unison, *i.e.* synchronous oscillations, of nonlinear systems [15]. This definition demands that all material points reach their extreme values and pass through zero simultaneously. Kerschen *et al.* extended the Rosenberg’s definition of NNMs to not-necessarily synchronous, periodic motions of the underlying undamped and unforced system [16]. An appealing feature of NNMs is that they can be used to predict approximately the frequency and the deformation shape of a nonlinear system at resonance, the quality of the approximation depending on the amount of damping in the system. A large body of literature addresses the qualitative and quantitative analyses of nonlinear phenomena using NNMs (the interested reader is referred to [17] for a review).

Recent publications reported the presence of isolas in frequency responses of nonlinear systems featuring 3:1 modal interactions between two of their NNMs [18, 19]. In [20], Kuether *et al.* formally established the link existing between modal interactions and isolas through an energy balance approach, and provided a numerical validation. As of today, however, no experimental demonstration of this theory has been proposed. Another observation is that, in the mechanical engineering literature, very few papers have been devoted to the experimental investigation of isolas. Gourc *et al.* found isolas while performing targeted energy transfer with a nonlinear energy sink [10]. In [21], remote attractors were detected in the response of an impacting pendulum. In [22], Gatti *et al.* analyzed the dynamics of a nonlinear oscillator attached to a shaker, and evidenced isolas in some experimental responses. In this context, the two objectives of the present paper are to realize isolas experimentally in a system featuring a modal interaction, and to relate isolas and modal interactions through a nonlinear modal analysis.

The paper is organized as follows. The Case Study section presents the illustrative mechanical application considered in the present work. It comprises two masses sliding on a horizontal guide and connected to the ground through linear and nonlinear springs. The Nonlinear Normal Modes section briefly reviews the relations between NNMs and nonlinear resonances, and introduces an energy balance criterion to predict isolated resonances. The experimental validation of the presented relations is achieved in the Experimental Realization section, where the responses of the two-mass test rig to swept-sine and stepped-sine excitations are studied to reveal isolas, and are compared to numerical simulations. The conclusions of the study are finally drawn in the last section of the paper.

## Case study: A 2-degree-of-freedom, base-excited, nonlinear system

In this section, the mechanical system considered throughout the paper for illustration and validation purposes is introduced, together with its experimental realization.

### Numerical representation

**Fig 1:**
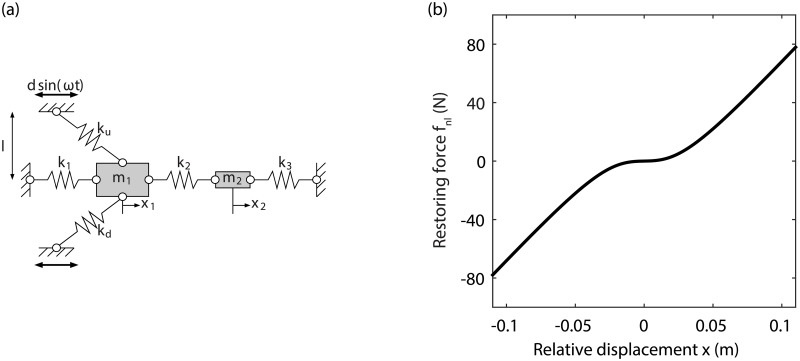
Illustrative mechanical system. (a) Schematic representation of the set-up; (b) numerical restoring force of the transverse connection.

Let us consider a system consisting of two masses connected through a linear spring and sliding on a horizontal guide, as shown in Fig 1(a). The physical and linear modal parameters of the system are listed in Tables 1 and 2, respectively. Two linear but transverse springs are also attached to mass 1, providing a nonlinear restoring force in the horizontal direction. The displacement of the transverse spring supports is prescribed to impart motion to the two masses. The mass and linear stiffness coefficients are such that a ratio between the linear natural frequencies of the system of 4.25, that is larger than 3 but slightly smaller than 5, is achieved. Moderate linear damping is finally introduced on the two vibration modes.

**Table 1:** Parameters of the numerical model.

*m*_1_ (kg)	*m*_2_ (kg)	*k*_1_ (N/m)	*k*_2_ (N/m)	*k*_3_ (N/m)
3.49	0.46	135.0	261.8	485.6
*k* (N/m)	*l* (m)	*λ* (-)	*c*_1_ (Ns/m)	*c*_2_ (Ns/m)
503.7	3.5 10^−2^	0.98	2.25	0.085

**Table 2:** Linear resonance frequencies and damping ratios computed from the numerical model.

Mode	Natural frequency (Hz)	Damping ratio (%)
1	1.52	3.33
2	6.46	0.24

The equations of motion of the system in Fig 1(a) can be derived as

$$\begin{array}{ccc} & & m_{1}{\ddot{x}}_{1}+c_{1}{\dot{x}}_{1}+k_{1}x_{1}+k_{2}(x_{1}-x_{2})+f_{nl}(x)=0\\ & & m_{2}{\ddot{x}}_{2}+c_{2}{\dot{x}}_{2}+k_{2}(x_{2}-x_{1})+k_{3}x_{2}=0 \end{array}$$ (1)

where *x*_1_ and *x*_2_ are the absolute displacements of *m*_1_ and *m*_2_, respectively, *x* = *x*_1_ − *d* sin(*ωt*) is the displacement of *m*_1_ relative to the imposed displacement of the base, *k*_1_, *k*_2_ and *k*_3_ are linear stiffness coefficients, and *c*_1_ and *c*_2_ are linear viscous damping coefficients. *f*_*nl*_ represents the horizontal projection of the restoring force associated with the two transverse springs. This force can be theoretically written as [23]

$$f_{nl}\left(x\right)=2F\text{cos}\left(\theta \right)=2k\left(\sqrt{x^{2}+l^{2}}-l_{0}\right)\;\frac{x}{\sqrt{x^{2}+l^{2}}}=2k\left(1-\frac{\lambda }{\sqrt{1+{\left(x/l\right)}^{2}}}\right)x$$ (2)

where *F* is the restoring force in the direction of a spring, *θ* is the angle between this direction and the horizontal, *k*, *l* and *l*_0_ denote the stiffness, length and natural length of the transverse chords, respectively, and *λ* = *l*_0_/*l* is the prestress parameter. The Taylor series expansion of Eq (2) around 0 reads

$$\begin{array}{c} f_{nl}\left(x\right)=2k\left(1-\lambda \right)x+\frac{k\lambda }{l^{2}}x^{3}+R_{3}\left(x\right) \end{array}$$ (3)

Eq (3) indicates the presence of a linear term in the restoring force that is related to the prestress of the chord, whereas the third-order term in the restoring force increases with *λ*. This confirms that limited prestress should be used to increase the nonlinear contribution in the transverse connection. The nonlinear force term in Eq (2) is depicted over a realistic displacement range in Fig 1(b).

The equations of motion (1) can be rewritten in order to obtain an external forcing vector **f**_*ext*_ (*ω*, *t*) equivalent to a harmonic base excitation *D* = *d* sin (*ωt*). This can be achieved by introducing new variables *x* = *x*_1_ − *D* and *y* = *x*_2_, yielding

$$\begin{array}{ll} m_{1}\ddot{x}+c_{1}\dot{x}+k_{1}x+k_{2}x-k_{2}y+f_{nl}\left(x\right) & =-m_{1}\ddot{D}-c_{1}\dot{D}-k_{1}D-k_{2}D\\ & =d\left(m_{1}\omega^{2}-k_{1}-k_{2}\right)\text{sin}\left(\omega t\right)-dc_{1}\omega \text{cos}\left(\omega t\right)\\ & =f_{ext,1}\\ m_{2}\ddot{y}+c_{2}\dot{y}+k_{2}y-k_{2}x+k_{3}y & =k_{2}D\\ & =dk_{2}\text{sin}\left(\omega t\right)\\ & =f_{ext,2} \end{array}$$ (4)

with

$$\begin{array}{c} \begin{array}{ccc} f_{ext}(\omega ,t)=d{\tilde{f}}_{ext}(\omega ,t), & & {\tilde{f}}_{ext}(\omega ,t)=[\begin{array}{c} \left(m_{1}\omega^{2}-k_{1}-k_{2})\text{sin}(\omega t)-c_{1}\omega \text{cos}(\omega t\right)\\ k_{2}\text{sin}(\omega t) \end{array}] \end{array} \end{array}$$ (5)

### Construction of the experimental set-up

**Fig 2:**
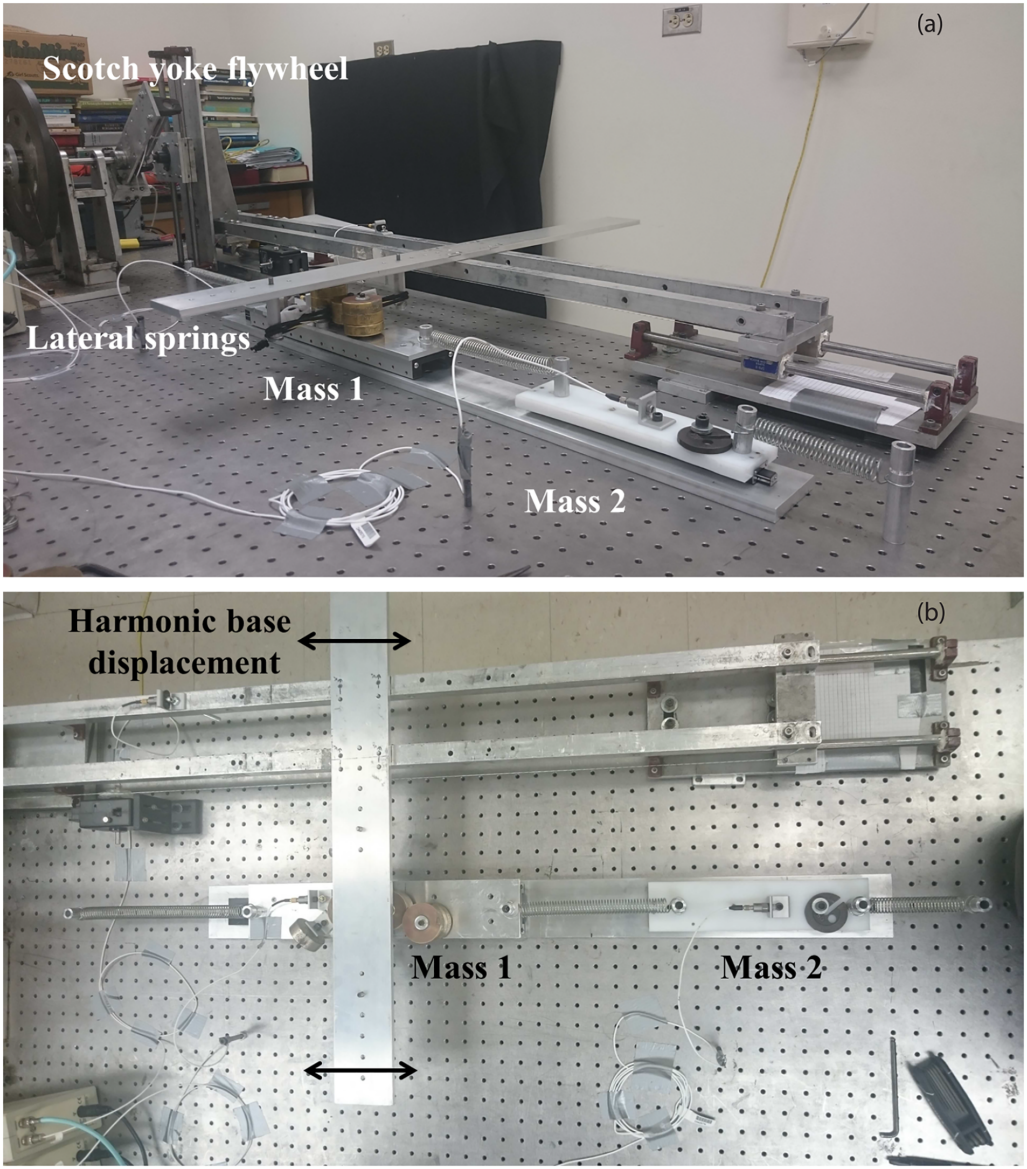
Experimental set-up. (a) Side view; (b) top view.

An experimental set-up featuring the same properties as the system described in the previous section was built at Duke University (NC, USA), and is shown in Fig 2. Two masses are connected together and to the ground through steel extension springs, whose lengths and stiffnesses determine the static equilibrium of the system. Two transverse bungee chords are attached to the first mass to provide a nonlinear restoring force in the direction of motion. All mass, stiffness and damping elements were adapted by trial and error in order to obtain similar properties as those of the numerical model presented in Tables 1 and 2.

The displacement of the transverse chord supports is prescribed to impart motion to the two masses using a Scotch yoke flywheel [23]. The Scotch yoke converts rotational motion into a unidirectional harmonic displacement *d* sin (*ωt*), where *d* is the base displacement amplitude, and *ω* the excitation frequency. The frequency *ω* is limited to 2.8Hz in the present experimental test rig, and the base displacement *d* is set manually but can be accurately estimated.

**Fig 3:**
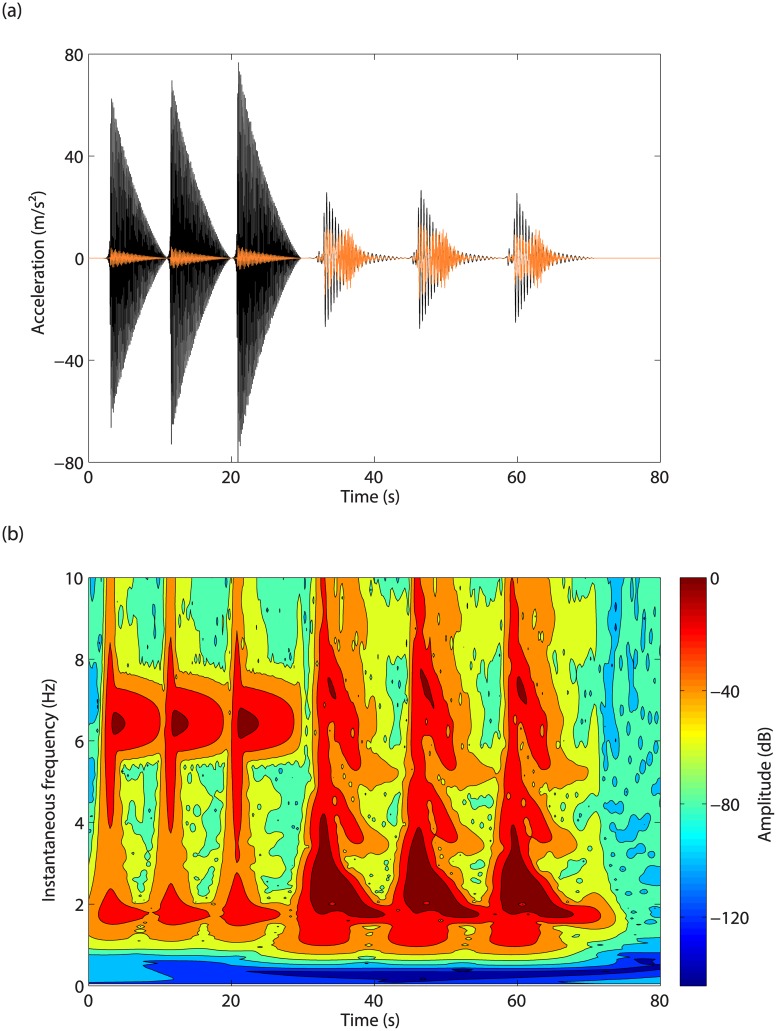
Free-decay analysis. (a) Accelerations measured on mass 1 (in black) and mass 2 (in orange) in response to three impacts applied to mass 2 followed by three impacts applied to mass 1; (b) wavelet transform of mass 1 acceleration.

Due to the limitation of the excitation frequency, the second mode of the system cannot be observed in forced conditions to verify its resonance frequency. Instead, the time series of free decays obtained after six consecutive impacts in Fig 3(a) are utilized to extract the resonance frequencies of the modes using the wavelet transform in Fig 3(b). The first mode exhibits a clear hardening, whereas the dependence on energy of the second mode is negligible. The resonance frequency of the second mode at low energy level is 6.45Hz; for the first mode, the resonance frequency is estimated around 1.5-1.6Hz.

Weak second and strong third harmonics of the first resonance frequency can also be observed in Fig 3(b). Interestingly, the intersection between the third harmonic of the first resonance frequency and the second resonance frequency involves an interaction, evidenced around 6.45Hz in Fig 3(b). This interaction, described in the next section as a modal interaction, is responsible for the distortion in the response of the second mass in Fig 3(a).

## Nonlinear normal modes and nonlinear resonances

The present section aims at introducing the fundamental relations between NNMs and nonlinear resonances. Readers interested in a comprehensive review of NNM properties are referred to [16, 24]. Let us consider the equations of motion of a *n*-degree-of-freedom (DOF) nonlinear systems

$$\begin{array}{c} M\ddot{x}+C\dot{x}+Kx+f_{nl}\left(x,\dot{x}\right)=f_{ext}\left(\omega ,t\right) \end{array}$$ (6)

where **M**, **C** and **K** are the mass, damping and stiffness matrices, respectively. Vectors **x**, **f**_*nl*_ and **f**_*ext*_ represent the displacements, the nonlinear forces and the external forces, respectively. **f**_*ext*_ is supposed herein to be periodic with frequency *ω*.

We define the frequency response of a nonlinear system as a branch of periodic solutions satisfying the equations of motion (6) for varying values of *ω*. The NNMs, on the other hand, are computed as branches of periodic solutions of the underlying undamped and unforced system, *i.e.*, periodic solutions of the Hamiltonian system

$$\begin{array}{c} M\ddot{x}+Kx+f_{nl}\left(x\right)=0 \end{array}$$ (7)

Note that the frequency responses and NNMs depicted in the paper were calculated using the combination described in [9] of a continuation procedure with the harmonic balance formalism. More specifically, each periodic solution was approximated by a Fourier series truncated to a certain order. In this work harmonics up the 9th order were retained, which could be shown to ensure convergence of the results.

### Properties of nonlinear normal modes

**Fig 4:**
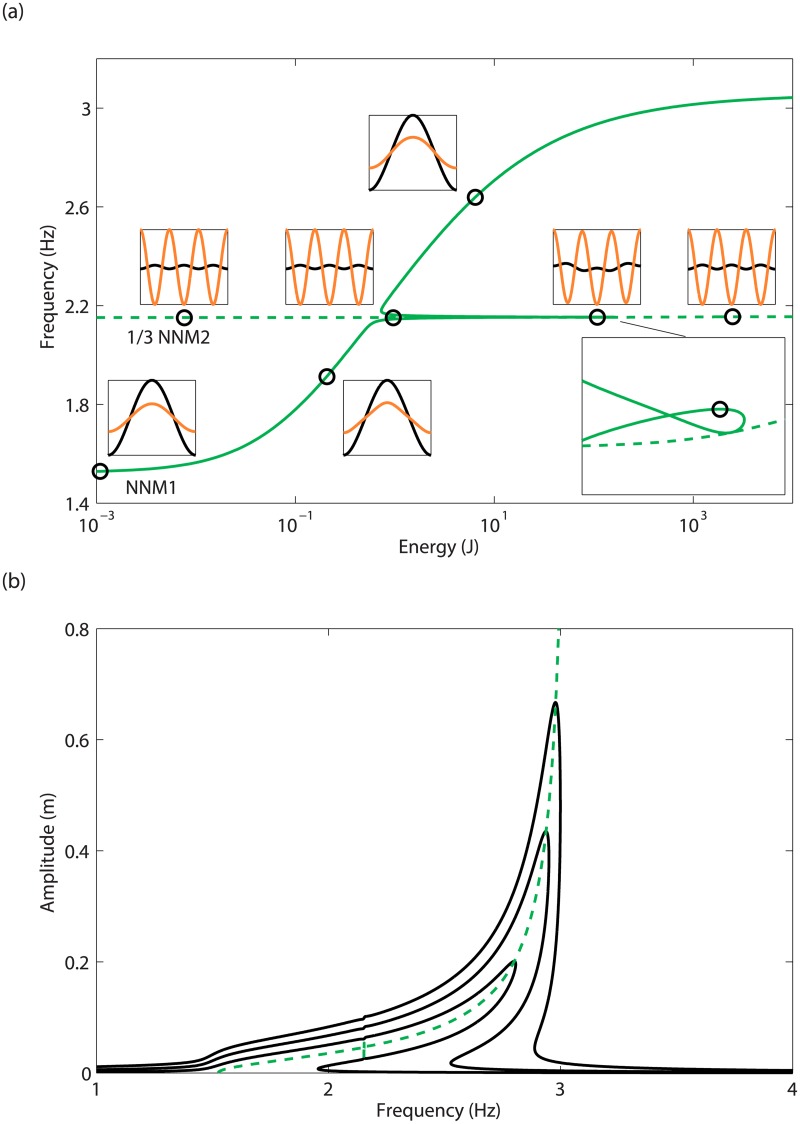
Fundamental properties of NNMs illustrated on the 2-DOF system. (a) FEP of the in-phase (solid line) NNM, and out-of-phase (dashed line) NNM represented at the third of its dominant frequency. NNM time series of mass 1 (in black) and mass 2 (in orange) are inset; (b) relation between the first NNM and the fundamental resonance. The solid lines represent the frequency responses computed at mass 1 for *d* = 10mm, *d* = 20mm and *d* = 30mm, and the dashed line depicts the projection of the first NNM in the frequency-response amplitude plane.

The fundamental properties of NNMs directly capture some features of nonlinear systems, as illustrated in Fig 4 on the 2-DOF numerical system introduced in the Case Study section.

#### Frequency-energy dependence

A peculiar property of nonlinear systems is the frequency-energy dependence of their oscillations, *e.g.*, through hardening or softening behaviors. The evolution of NNMs along their backbone accounts for this dependence, which can be conveniently depicted in a frequency-energy plot (FEP).

The continuation of NNMs is carried out on the 2-DOF system in Fig 4(a), where the FEP of the two branches related to the first and second modes is depicted. The second NNM is represented at the third of its dominant frequency, which is relevant because a periodic solution of period *T* is also periodic with period 3*T*. The energy dependence is strong for the first NNM and negligible for the second one. Regarding the mode shapes, the in-phase NNM mostly involves a motion of mass 1, except near 2.15Hz. Conversely, the shape of the out-of-phase NNM is barely affected by the energy level, and exhibits larger oscillations of mass 2.

#### Modal interactions

When progressing along the NNM backbone, harmonics of the fundamental frequency are generated by the nonlinearities, and may have a frequency close to the oscillation frequency of another NNM of the system. In this situation, referred to as a modal interaction or an internal resonance, a dynamic coupling between the two modes is established together with an energy transfer. Due to the frequency-energy dependence of NNMs, such interactions can develop between modes with non-commensurate linear frequencies.

In Fig 4(a), the branch of the first NNM crosses the branch of the second NNM which dominant frequency was divided by 3. This indicates that near 2.15Hz, the contribution of the third harmonic generated along the first NNM can internally excite the second NNM branch. The first and second NNMs thus start exchanging energy through a 3:1 modal interaction in this region, translating into the appearance of a new topological feature called a tongue.

#### Relations with forced responses

For structures with low damping, the NNM backbone traces the locus of the nonlinear resonance peaks. This feature is illustrated in Fig 4(b), which compares the first resonance peak of the 2-DOF system at different levels *d* of base displacement, with the projection of the first NNM in the frequency-response amplitude plane.

### Energy balance formulation

From Fig 4(b), it is clear that the resonant response of a structure can be characterized through the excitation of the corresponding NNM. As a consequence, recent efforts have been devoted to determine the parameters of the forcing that appropriates a given NNM. A first analytical attempt in this direction [25, 26] used the second-order normal form theory to develop a nonlinear extension of the energy balance criterion [14]. Because this method assumes weak nonlinearity, Kuether *et al.* proposed in [20] a numerical formulation of the energy balance to tackle strongly nonlinear regimes of motion.

Let us consider a nonlinear system that oscillates in a NNM denoted as **x**(*t*). The damping forces instantaneously exert a distributed force $C\dot{x}\left(t\right)$ and the total energy dissipated over one period of oscillation *T* is

$$\begin{array}{c} E_{diss}=\int_{0}^{T}P_{diss}\;dt=\int_{0}^{T}\dot{x}{\left(t\right)}^{\text{T}}C\dot{x}\left(t\right)\;dt \end{array}$$ (8)

where *P*_*diss*_ is the power dissipated at any instant. Similarly, an arbitrary harmonic forcing function **f**_*ext*_(*t*) inputs energy into the system as

$$\begin{array}{c} E_{in}=\int_{0}^{T}P_{in}\;dt=\int_{0}^{T}\dot{x}{\left(t\right)}^{\text{T}}f_{ext}\left(t\right)\;dt \end{array}$$ (9)

where *P*_*in*_ is the power input into the system at any instant. At resonance, since the response **x**(*t*) verifies both Eqs (6) and (7), the energy dissipated by the damping forces must match the total energy input to the system over the period *T* [14]. The balance is enforced by setting

$$\begin{array}{c} E_{diss}=E_{in} \end{array}$$ (10)

Considering the more practical case of a monoharmonic force applied to a single DOF *l*, **f**_*ext*_(*t*) = *f* **e**_*l*_ sin (*ωt*), where **e**_*l*_ is a *n* × 1 vector of zeros with a value of one at the component *l*, and hence

$$\begin{array}{c} P_{in}=\dot{x}{\left(t\right)}^{\text{T}}fe_{l}\text{sin}(\omega t) \end{array}$$ (11)

The energy balance for a mono-point, monoharmonic forcing can be finally expressed as

$$\begin{array}{c} f=\frac{\int_{0}^{T}\dot{x}{\left(t\right)}^{\text{T}}C\dot{x}\left(t\right)\;dt}{\int_{0}^{T}\dot{x}{\left(t\right)}^{\text{T}}e_{l}\text{sin}(\omega t)\;dt} \end{array}$$ (12)

The relation (12) stands for an important result because, given a forcing frequency *ω*, a specific NNM **x**(*t*) and the damping matrix **C**, it estimates the forcing amplitude *f* that excites the system at resonance with the associated NNM motion.

In the context of the present work, the relation (10) can be rewritten using Eqs (4) and (5) to assess the evolution of the nonlinear resonances with respect to the base displacement *d*, yielding

$$\begin{array}{c} d=\frac{\int_{0}^{T}(c_{1}{\dot{x}}_{1}^{2}\left(t\right)+c_{2}{\dot{x}}_{2}^{2}\left(t\right))\;dt}{\int_{0}^{T}({\dot{x}}_{1}\left(t\right){\tilde{f}}_{ext,1}\left(\omega ,t\right)+{\dot{x}}_{2}\left(t\right){\tilde{f}}_{ext,2}\left(\omega ,t\right))\;dt} \end{array}$$ (13)

### Prediction of fundamental and isolated resonances

**Fig 5:**
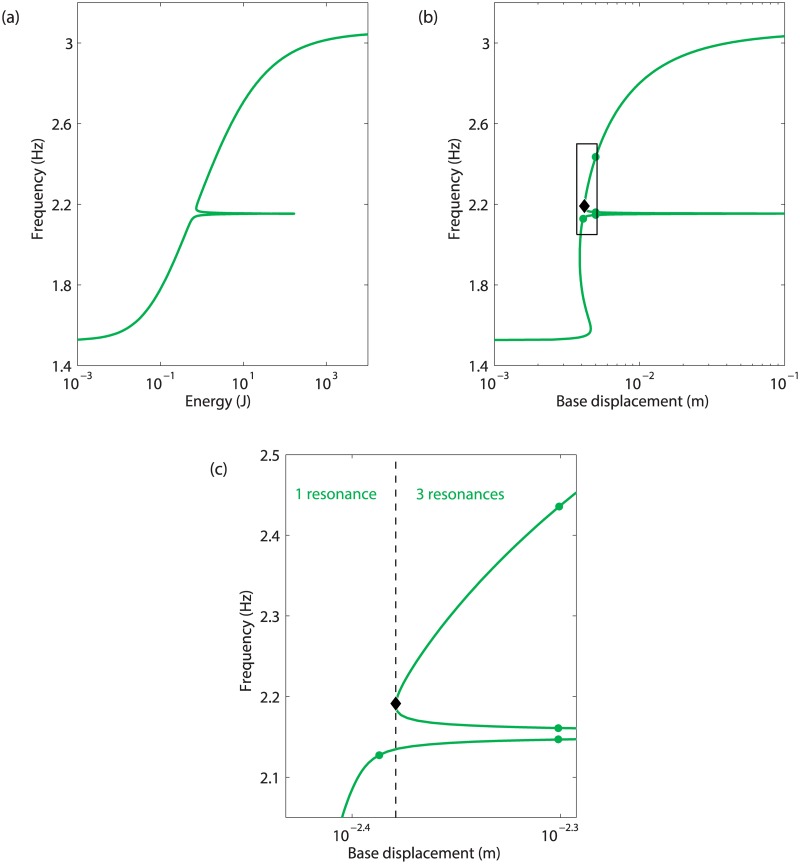
Energy balance applied to the first NNM of the 2-DOF system. (a) FEP of the NNM; (b) estimate of the base displacement required to obtain the motion given at each point on the first NNM. The dots indicate the resonances at *d* = 4.1mm and 5mm, and the diamond marker locates the creation of new resonances due to the 3:1 modal interaction; (c) close-up of (b).

The application of the energy balance Eq (13) to the first NNM of the system in Eq (4) is depicted in Fig 5. If only one resonance can be excited at low base displacements, the folding of the curve in Fig 5(b), denoted by the diamond marker, results in the appearance of two additional resonances beyond *d* = 4.2mm. The presence of this folding is due to the existence of a modal interaction, near 2.15Hz.

**Fig 6:**
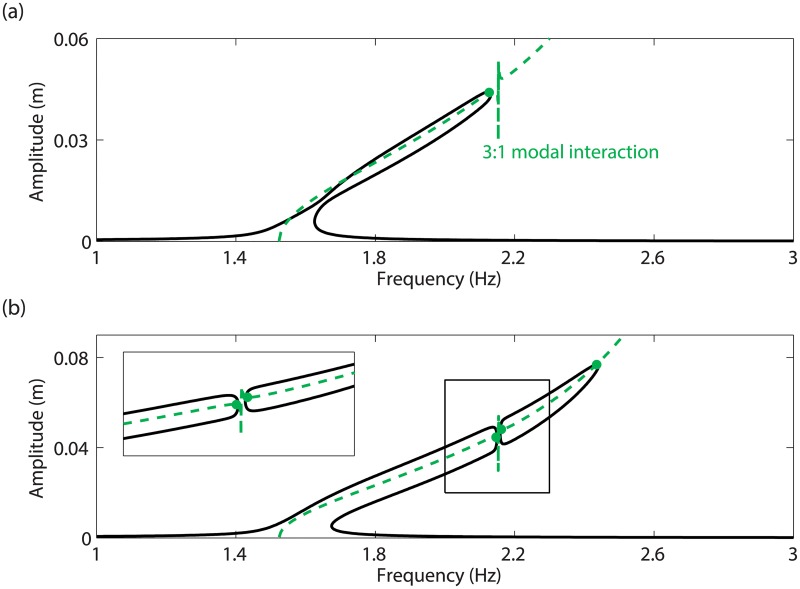
Comparison between the frequency responses at mass 1 and the NNM motions determined from the energy balance criterion in Fig 5(b) (dots). (a) *d* = 4.1mm; (b) *d* = 5mm and close-up. The projection of the first NNM in the frequency-response amplitude plane is plotted as a dashed line.

In order to investigate the impact of the additional resonances on the forced response of the system, Fig 6(a) and 6(b) represent the frequency responses computed at *d* = 4.1mm and *d* = 5mm, respectively. The projection of the NNM curve in the frequency-response amplitude plane, and the NNM motions detected as resonances in Fig 5(b) are also shown. Fig 6(a) confirms that only one resonance related to the main peak can be found; this fundamental resonance is accurately detected along the NNM curve. In Fig 6(b), *i.e.*, after the appearance of the two additional resonances, the main resonance peak is accompanied with an isolated branch of periodic solutions, also called isola, with isolated resonances at its extremities. It is stressed that isolas cannot be computed directly from the resonance peak using classical continuation techniques, and that the bifurcation continuation algorithm described in [9] had to be employed. Therefore isolas most often remain undetected despite their possibly large amplitude. This illustrates the importance of the prediction capabilities of the energy balance method to reveal not only fundamental but also isolated resonances.

**Fig 7:**
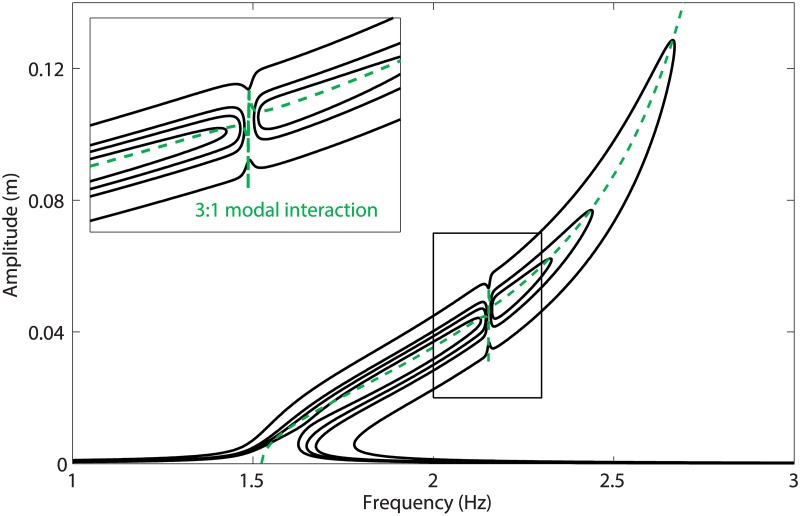
Merging of the isola with the main frequency response, and close-up. The solid lines represent the frequency responses computed at mass 1 for *d* = 4:1 mm, *d* = 4:5 mm, *d* = 5mm and *d* = 7 mm, and the dashed line depicts the projection of the first NNM in the frequency-response amplitude plane.

As the base displacement *d* is increased, the isola expands both in frequency and amplitude. It eventually merges with the resonance peak in the vicinity of the 3:1 modal interaction, for a value of *d* between 5mm and 7 mm, as illustrated in Fig 7. The energy balance curve in Fig 5(b), on the other hand, shows around 2:2 Hz the merging of the fundamental resonance and of one of the isolated resonances for a base displacement larger than 100 mm. This discrepancy between the energy balance prediction and the numerical continuation can be explained by the fact that the merging phenomenon is strongly affected by the damping forces, and involves higher harmonics of the fundamental frequency that are not considered in the energy balance.

## Experimental realization of isolas

The results in the Nonlinear Normal Modes section confirm the important role played by modal interactions in the creation of isolas, highlighted by the energy balance formalism. The purpose of the present section is to validate experimentally these theoretical findings using the experimental set-up described in the Case Study section.

### Detection of isolas in forced responses

**Fig 8:**
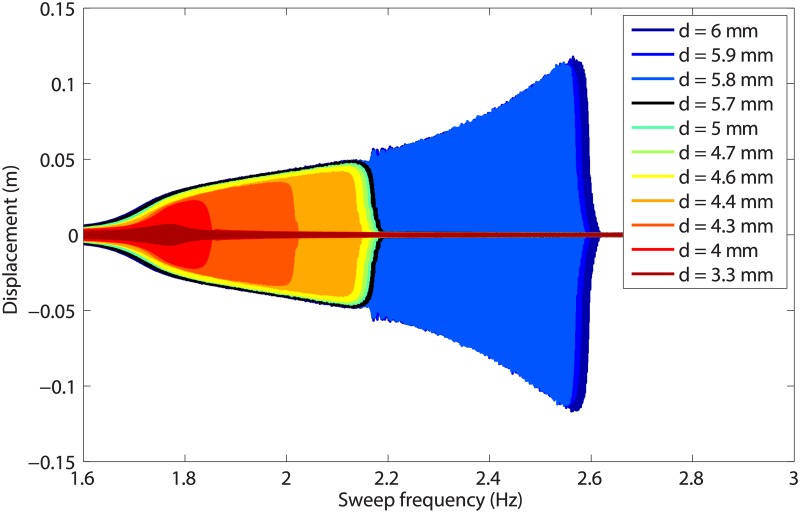
Responses of mass 1 to swept-sine excitations for different values of the base displacement *d*.

**Fig 9:**
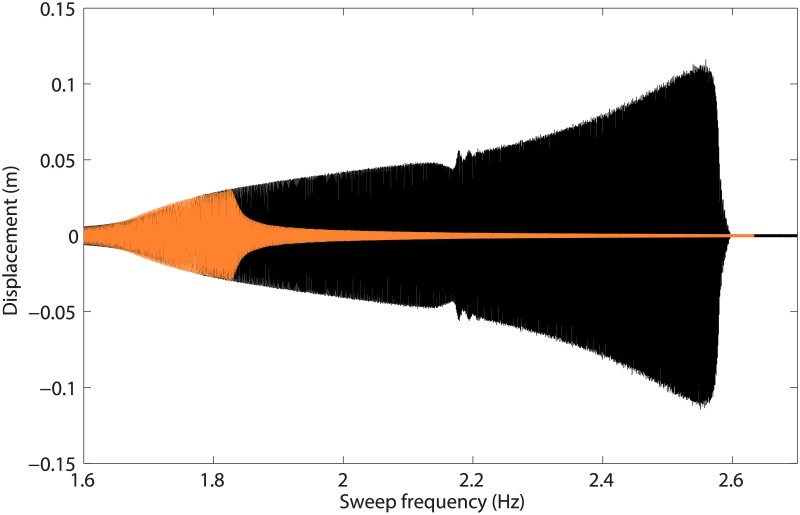
Response of mass 1 to swept-sine excitations with positive (in black) and negative (in orange) sweep rates, for *d* = 5.9mm.

The approach followed in this work to reveal the presence of isolas is to record responses to swept-sine excitations of increasing amplitude. This is carried out in Fig 8 for values of *d* ranging from 3.3mm to 6mm. When *d* = 3.3mm, the geometrical nonlinearity at the first mass is not activated, and the dynamics is predominantly linear. From *d* = 4mm to 5.7mm, there is an increase in both amplitude and frequency at resonance, which is followed by the classical jump to low-amplitude solution. As *d* becomes closer to 5.7mm, the resonance frequency saturates near 2.15Hz. Increasing *d* from 5.7mm to 5.8mm leads to a dramatic increase (18.6%) in the resonance frequency from 2.15Hz to 2.55Hz. This mechanism, *i.e.*, the saturation of the resonance frequency followed by its sudden shift, is often observed for systems featuring modal interactions [20, 27]. At that point, a mechanism similar to the one in Fig 7, *i.e.*, the merging of the main frequency response with an isola, is suspected. Fig 9 depicts the sweep-up and sweep-down responses of mass 1 for a base amplitude of 5.9mm, *i.e.*, after the suspected merging of the isola. Jumps down and up occur around 2.55Hz and 1.8Hz, respectively, implying that the system possesses a very large bistable region.

**Fig 10:**
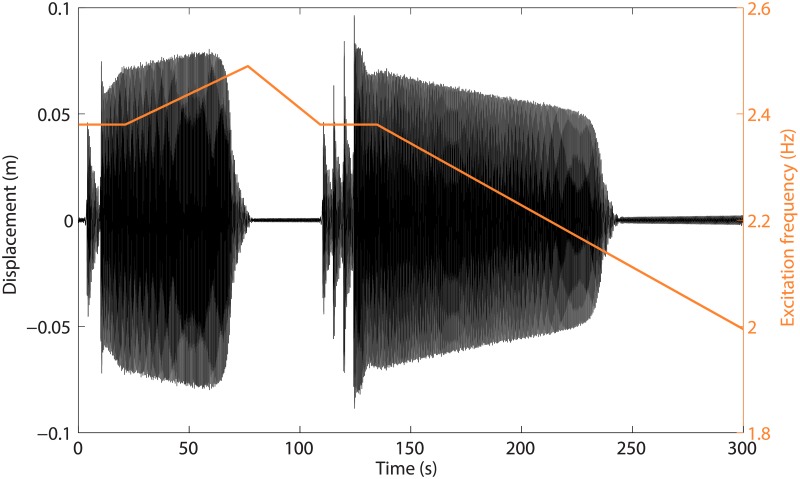
Realization by perturbations of periodic solutions on the detached isola. The amplitude and initial frequency of the base excitation are *d* = 5mm and *ω* = 2.38Hz, respectively. The displacement of mass 1 and the excitation frequency are displayed through black and orange lines, respectively.

To bring experimental evidence of the isola, time series corresponding to motions on the detached branch are depicted in Fig 10. To realize these time series, a base excitation of 5mm and 2.38Hz is considered; according to the results in Fig 8, this frequency is located beyond the main resonance peak. The system initially vibrates at low amplitude, *i.e.*, on the main frequency response branch, and a series of perturbations is applied to excite the isola. Around *t* = 10s, a high-amplitude motion stabilizes, confirming the existence of the isola. From there on, a sweep up followed by a jump around *t* = 70s locates the right extremity of the stable portion of the isola at 2.47Hz. Between *t* = 70s and *t* = 110s, the excitation is swept back to 2.38Hz, when a new series of perturbations is applied. At *t* = 125s, the system is back on the isola, and a sweep down permits to travel along it until *t* = 240s. This new jump locates the left extremity of the stable portion of the isola at 2.16Hz. Since the basin of attraction of the isolated response shrinks as the limits of its stable part are reached, it may have happened that the system jumped before the actual change in stability. A solution to this problem would be to perform stochastic interrogation in the frequency range of interest [23] or experimental continuation [28, 29], but this was beyond the scope of the present work.

### Bifurcation analysis near the modal interaction

**Fig 11:**
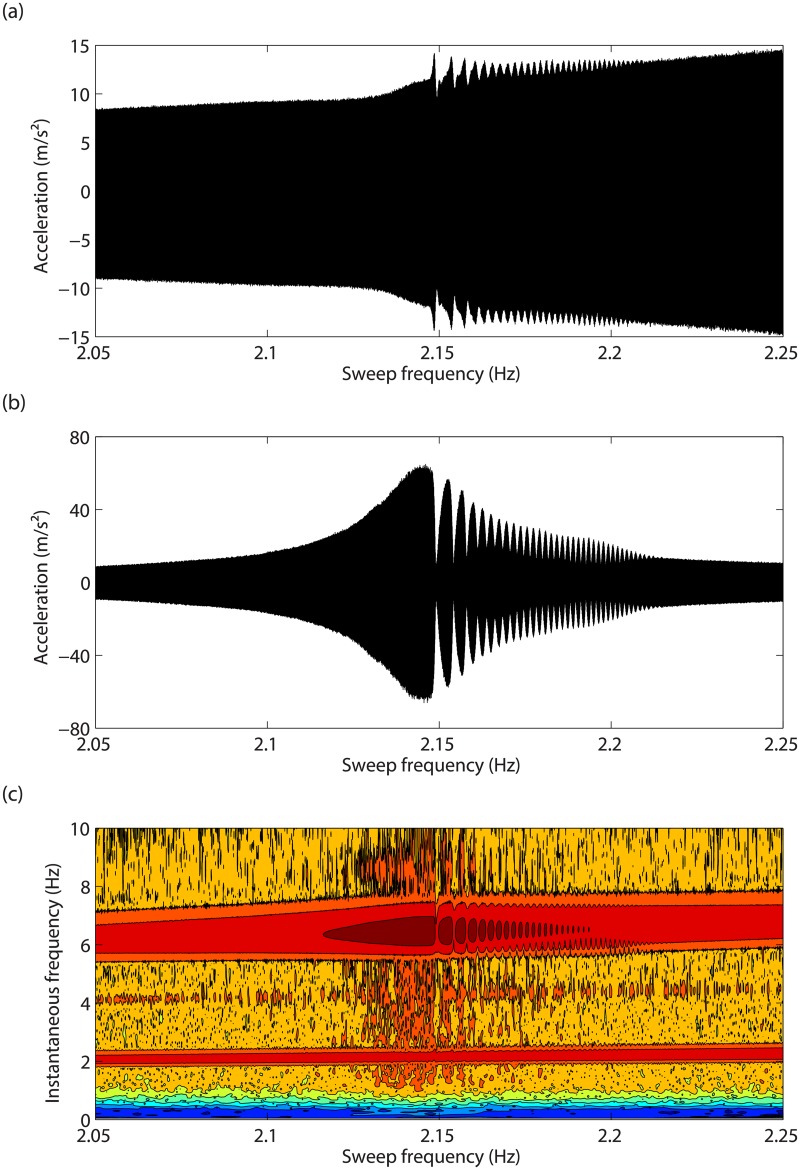
Close-up on the merging region. Accelerations of (a) mass 1 and (b) mass 2, and (c) wavelet transform of mass 2 acceleration in response to swept-sine excitations for *d* = 5.9mm.

As observed in Fig 8, the main resonant response saturates near 2.15Hz before the merging. Since 2.15Hz is precisely the third of the resonance frequency of the second mode, it is clear that the merging process takes place in the vicinity of the 3:1 modal interaction between the in-phase and out-of-phase modes. This experimental result is thus the confirmation that modal interactions are one possible dynamical mechanism for the creation of isolas. A closer investigation after merging is carried out in Fig 11, which shows acceleration signals measured on the two masses near 2.15Hz for *d* = 5.9mm. A strong modulation of the time series is observed in Fig 11a and 11b. The wavelet transform in Fig 11(c) reveals the presence of a significant third harmonic component, the frequency of which coincides with the frequency of the out-of-phase mode. We refer the interested reader to S1 and S2 Videos for videos of the swept-sine responses recorded near the merging region for the isolated (*d* = 5.7mm) and merged (*d* = 6mm) configurations, respectively. Both videos highlight the contributions of the third harmonic of the excitation frequency.

**Fig 12:**
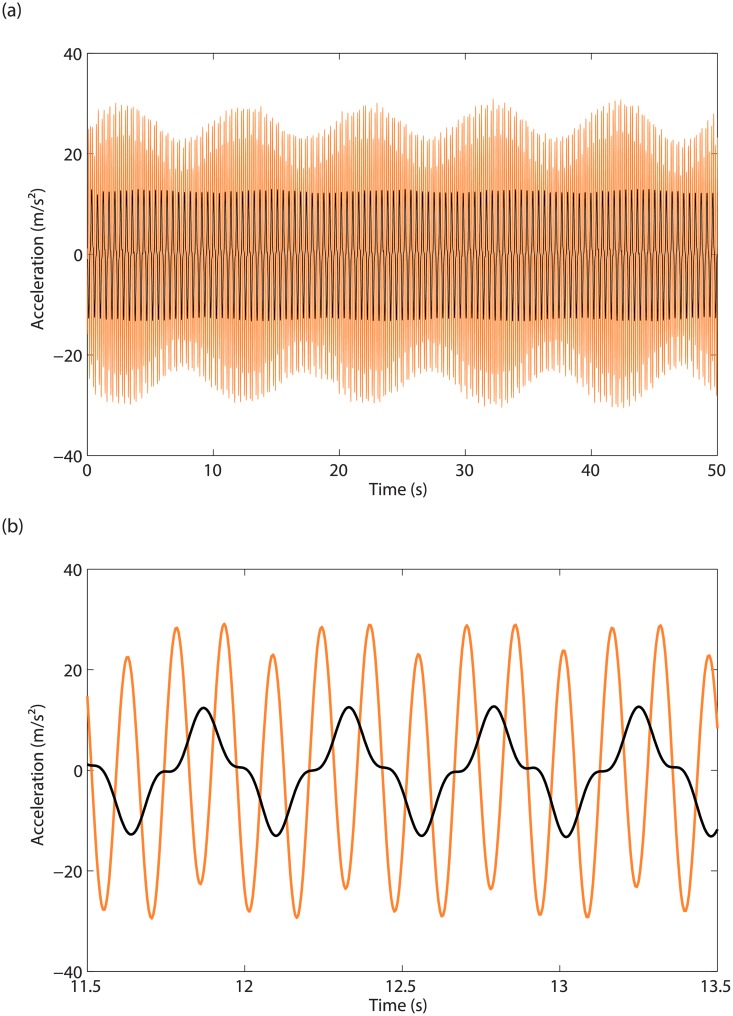
Evidence of the 3:1 modal interaction. (a) Time series for the acceleration of mass 1 (in black) and 2 (in orange), for *d* = 5.9mm and *ω* = 2.17Hz; (b) close-up.

The response of the two masses to a stepped-sine excitation at 2.17Hz is displayed in Fig 12. The 3:1 modal interaction is clearly visible in Fig 12(b), whereas the modulation observed in Fig 11 is repeated in Fig 12(a), which seems to relate to quasiperiodic oscillations with an envelope frequency of 0.1Hz.

### Correlation with the numerical model

**Fig 13:**
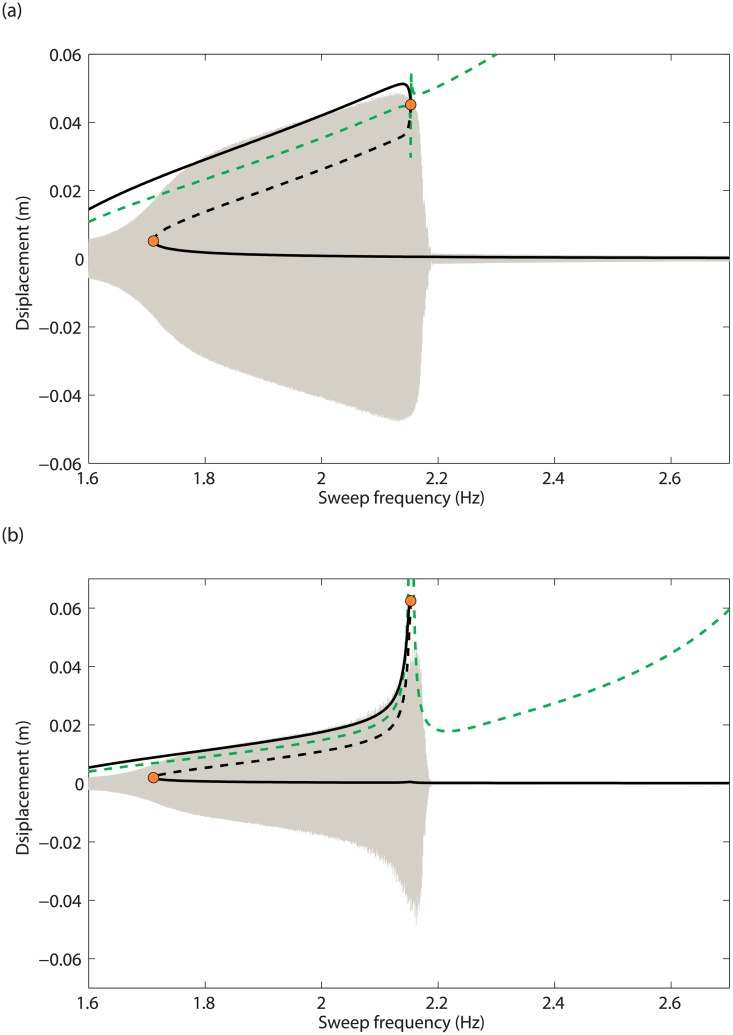
Comparison between the experimental swept-sine response (in grey), the backbone of the first NNM (in green) and the frequency response (in black), before the merging, for *d* = 5.7mm. (a) Mass 1; (b) mass 2. Solid and dashed line represent stable and unstable solutions along the frequency response, respectively. Fold bifurcations are signaled via circle markers.

**Fig 14:**
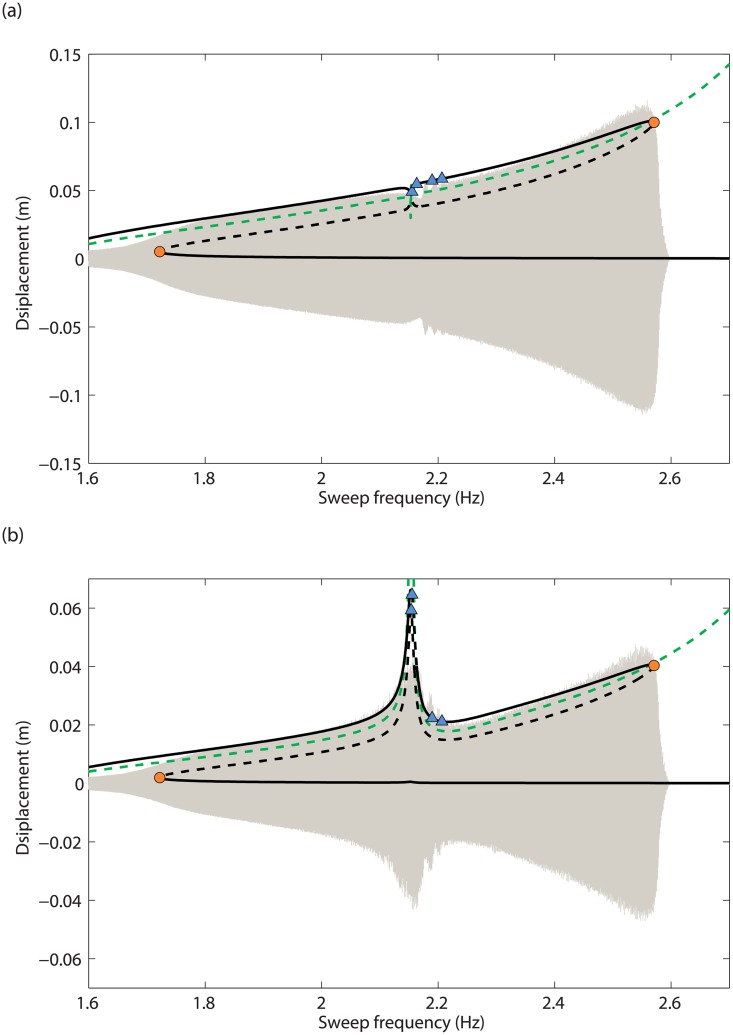
Comparison between the experimental swept-sine response (in grey), the backbone of the first NNM (in green) and the frequency response (in black), after the merging, for *d* = 5.9mm. (a) Mass 1; (b) mass 2. Solid and dashed line represent stable and unstable solutions along the frequency response, respectively. Fold and NS bifurcations are signaled via circle and triangle markers, respectively.

In Figs 13 and 14, the frequency responses of the model in Eq (1) and their bifurcations are compared to the experimental swept-sine responses, for *d* = 5.7mm and *d* = 5.9mm, respectively. For the sake of clarity, the isola is not represented in the former case. The superposition of the frequency responses and the experimental time series demonstrate the capabilities of the model to capture the dynamics of the system, especially in the merging region. In particular, the sudden increase in the displacement of mass 2 in the vicinity of the 3:1 modal interaction is represented, which can be attributed to the energy exchange between the in-phase and out-of-phase modes. On the other hand, the discrepancies for the amplitudes of the two masses at the 3:1 modal interaction, at resonance and at low amplitude can be attributed to the inaccurate estimation of the damping forces.

**Fig 15:**
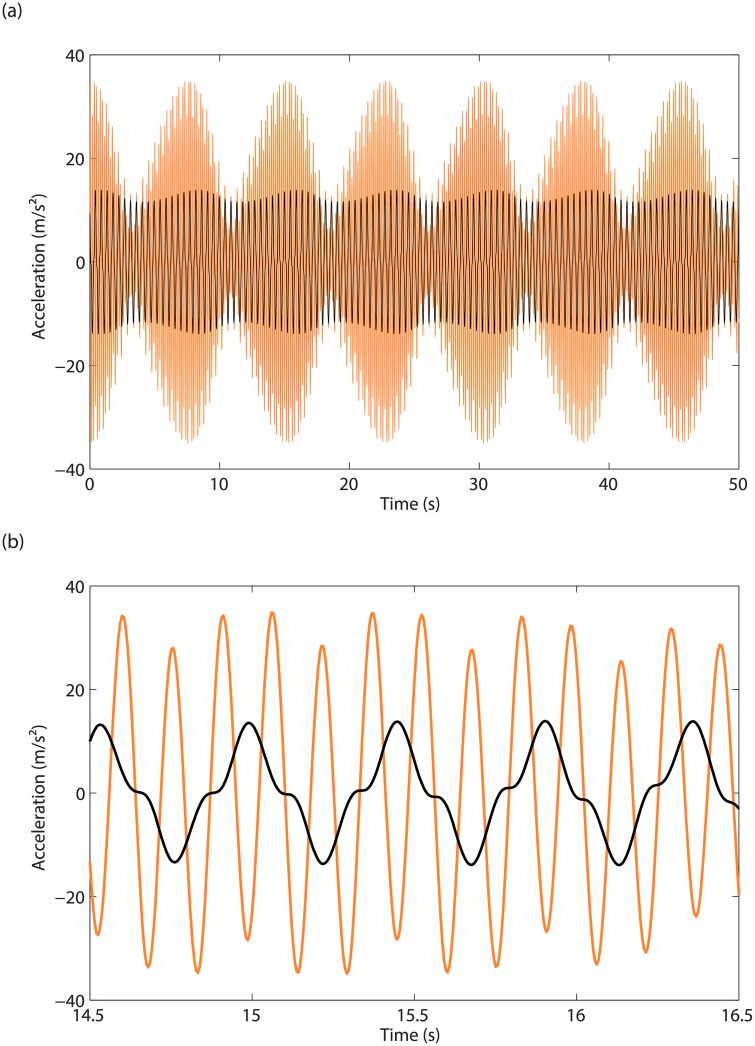
Quasiperiodic oscillations obtained from time integration. (a) Time series for the accelerations of masses 1 (in black) and 2 (in orange), for *d* = 5.9mm and *ω* = 2.196Hz; (b) close-up.

Neimark-Sacker (NS) bifurcations are detected in the merging region in Fig 14, and could explain the modulated oscillations observed in Fig 12. In Fig 15, time integration is performed at 2.196Hz in the unstable region between the third and fourth NS bifurcations. Stable quasiperiodic oscillations are obtained, which bare strong resemblance with the experimental time series presented in Fig 12.

### Influence of the modal interaction

**Fig 16:**
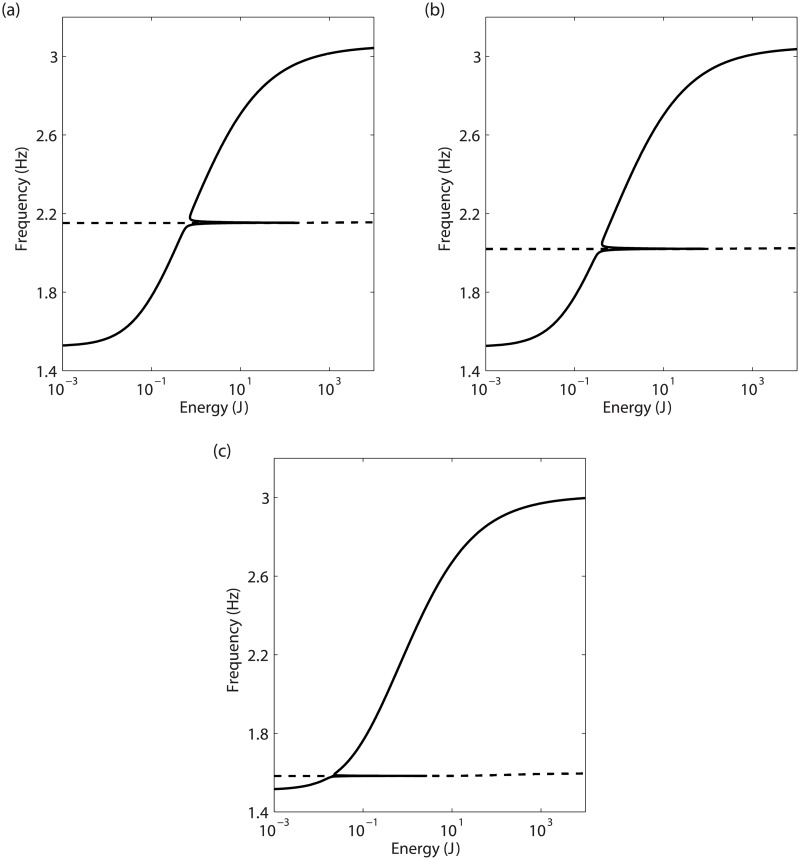
Influence of *m*_2_ on NNM 1 (solid line), and NNM 2 represented at the third of its dominant frequency (dashed line). (a) *m*_2_ = 0.464kg (reference); (b) *m*_2_ = 0.526kg; (c) *m*_2_ = 0.868kg.

Experiments and simulations confirmed that the presence of the isola is closely related to the 3:1 modal interaction. Let us now investigate the effects of the modification of the modal interaction location. To this end, the NNMs computed for several values of the parameter *m*_2_ are displayed in Fig 16. Increasing the value of *m*_2_ is shown to have a limited influence on the first mode, but a strong impact on the second mode, which natural frequency decreases. This involves, in turn, a shift of the 3:1 modal interaction to lower frequencies.

**Fig 17:**
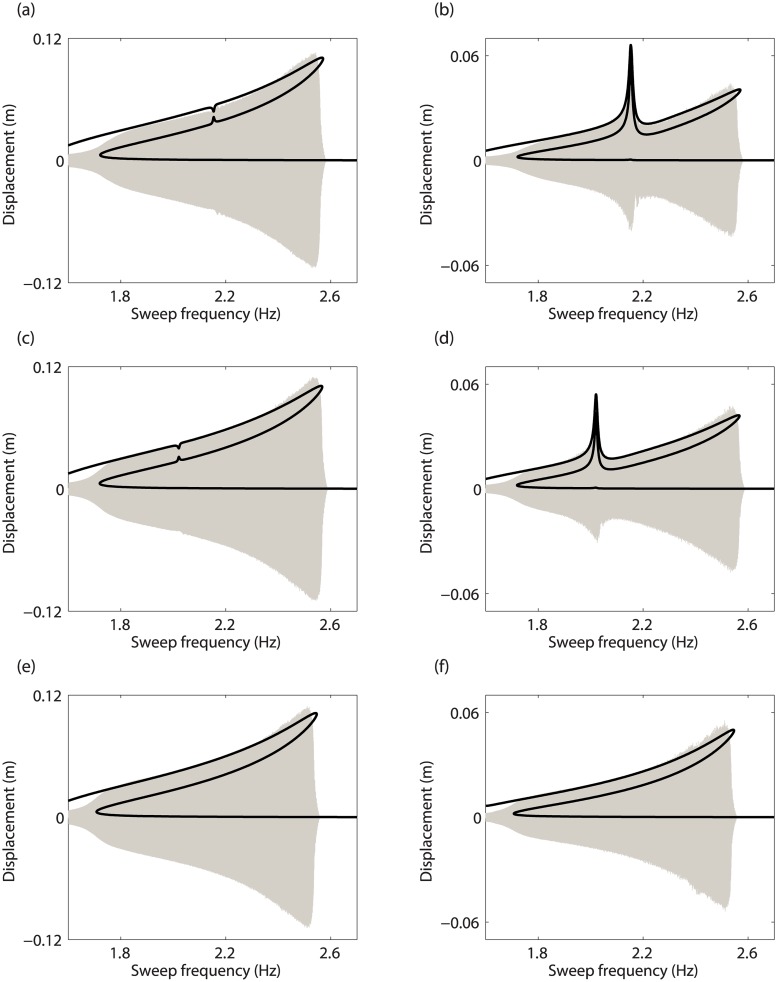
Influence of *m*_2_ on the forced response of mass 1 (left column) and mass 2 (right column), for *d* = 5.9mm. (a-b) *m*_2_ = 0.464kg (reference); (c-d) *m*_2_ = 0.526kg; (e-f) *m*_2_ = 0.868kg. The experimental swept-sine responses and the numerical frequency responses are represented with grey and black lines, respectively.

The influence of the modal interaction on the responses of the system is studied both experimentally and numerically in Fig 17. For the reference configuration in Fig 17a and 17b, the merging of the isola occurs around 2.15Hz. When *m*_2_ is augmented up to 0.526kg, the modal interaction is detected at 2.02Hz (see Fig 17c and 17d). For this configuration, the isola still exists. Increasing *m*_2_ to 0.868kg eventually leads to the disappearance of the 3:1 modal interaction from the frequency response in Fig 17e and 17f.

**Fig 18:**
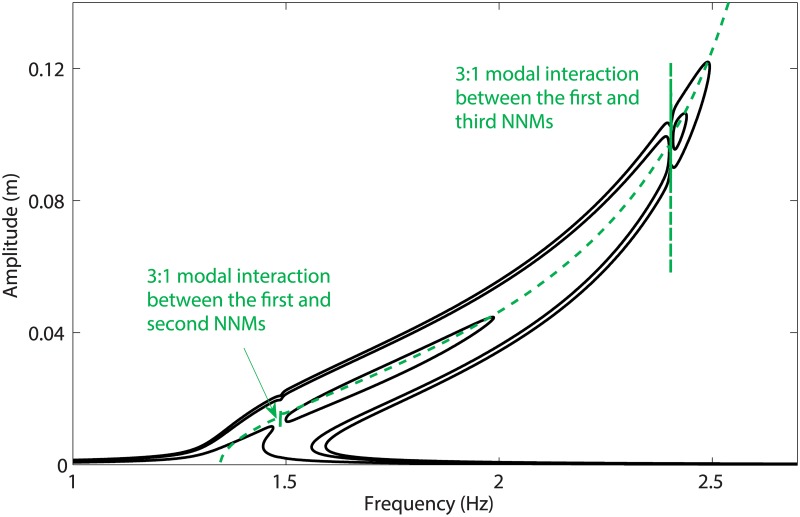
Double isola scenario observed for a 3-DOF model. The solid lines are the frequency responses computed for *d* = 3.8mm, *d* = 5.9mm and *d* = 6.5mm, and the dashed line shows the projection of the first NNM in the frequency-response amplitude plane.

As a closing result, Fig 18 illustrates the frequency responses of a 3-DOF model consisting in the previous 2-DOF system to which a third mass of 0.5kg is added. Although an exhaustive description of the dynamical behavior of the 3-DOF system is beyond the scope of this work, Fig 18 shows that increasing the base displacement for this set-up leads to two consecutive sequences of creation of an isola followed by its merging with the main frequency response. Interestingly, the first NNM of this system features 3:1 interactions with the second and third NNM, and both merging occur near these modal interactions.

## Conclusions

This paper provided numerical and experimental evidence for the presence of isolated resonances in the vicinity of modal interactions. First, nonlinear modal and frequency response analyses carried out on the numerical model of a 2-degree-of-freedom system revealed the appearance of an isola and its merging with the main response near a 3:1 modal interaction. These simulations also shed light on the remarkable capabilities of the energy balance approach to predict multiple resonance scenarios.

The numerical observations could be accurately reproduced using an experimental set-up. The stable portion of an isola and its merging with the fundamental resonance branch were carefully characterized combining swept-sine and stepped-sine excitations. As a consequence of the merging, the isola was found to be responsible for a sudden and substantial increase in the resonance frequency of the in-phase mode.

Overall, this work validated the theoretical finding of [20] that modal interactions are possible mechanisms for creating isolas, and offered a better understanding of isolas and their influence on the nonlinear system dynamics.

## Supporting information

S1 Video Isolated configuration. Video of the experimental swept-sine response obtained for a base displacement *d* = 5.7mm.
(MP4)

S2 Video Merged configuration. Video of the experimental swept-sine response obtained for a base displacement *d* = 6mm.
(MP4)
